# Exchange Bias in Nanostructures: An Update

**DOI:** 10.3390/nano13172418

**Published:** 2023-08-25

**Authors:** Tomasz Blachowicz, Andrea Ehrmann, Martin Wortmann

**Affiliations:** 1 Institute of Physics—Center for Science and Education, Silesian University of Technology, ul. Konarskiego 22B, 44-100 Gliwice, Poland; tomasz.blachowicz@polsl.pl; 2 Faculty of Engineering and Mathematics, Bielefeld University of Applied Sciences and Arts, Interaktion 1, 33619 Bielefeld, Germany; 3 Faculty of Physics, Bielefeld University, Universitätsstraße 25, 33615 Bielefeld, Germany; mwortmann@physik.uni-bielefeld.de

**Keywords:** exchange bias (EB), hysteresis loop shift, coercivity, ferromagnet, antiferromagnet, coercive field, asymmetric hysteresis loop

## Abstract

Exchange bias (EB) is a unidirectional anisotropy occurring in exchange-coupled ferromagnetic/antiferromagnetic systems, such as thin films, core–shell particles, or nanostructures. In addition to a horizontal shift of the hysteresis loop, defining the exchange bias, asymmetric loops and even vertical shifts can often be found. While the effect is used in hard disk read heads and several spintronics applications, its origin is still not fully understood. Especially in nanostructures with their additional shape anisotropies, interesting and often unexpected effects can occur. Here, we provide an overview of the most recent experimental findings and theoretical models of exchange bias in nanostructures from different materials.

## 1. Introduction

The exchange bias (EB), a unidirectional magnetic anisotropy, was first reported by Meiklejohn and Bean for Co/CoO core–shell particles [1,2] and has since been extensively investigated. The main effect is a horizontal shift of the hysteresis loop in a system consisting of a ferromagnet (FM) exchange-coupled to an antiferromagnet (AFM), similar to a frozen internal magnetic field applied to the FM by the AFM’s fixed magnetic moments. Nevertheless, this simple explanation and corresponding naïve models cannot fully simulate the value of the horizontal shift, nor the other correlated changes in the hysteresis loop as compared to pure ferromagnets, i.e., an often-visible asymmetry of the loop as well as a potential vertical shift [3].

In addition to the first investigations of core–shell particles, usually with a ferromagnetic core and oxidized antiferromagnetic shell, experiments have evolved rapidly towards thin film systems [4,5] and further to nanostructured systems [6,7,8]. The materials under investigation are often ferromagnets coupled with antiferromagnets or ferrimagnets, such as Co/CoO [9], Fe/FeF_2_ [10], or Fe/MnF_2_ [11,12]. Recently, more sophisticated systems have become part of experimental and theoretical studies, e.g., Fe/LaAlO_3_ [13] or Pr_0.67_Sr_0.33_MnO_3_/SrTiO_3_ [14].

**Figure 1:**
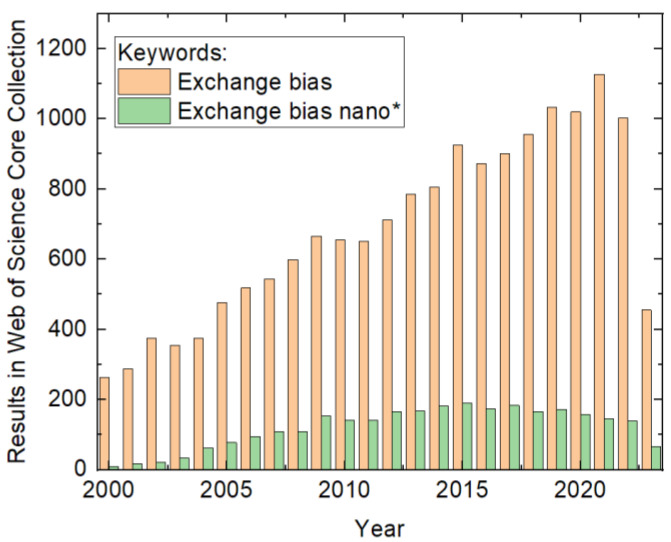
Numbers of results in the Web of Science Core Collection for the keywords provided in the inset, counted on 15 July 2023.

In addition to developing new materials for innovative EB systems with an enhanced effect size, more asymmetric loops, or other technically useful effects, e.g., applications for hard disk read/write heads and spintronics devices, the magnetic properties of well-known material systems can also be manipulated by creating nanostructures with different shapes and dimensions. In their comprehensive review from 2005, Nogués et al. described the effects in detail of the former state of research [6]. However, research activities dealing with exchange bias in general and exchange bias in nanostructures in particular have considerably increased since then, as depicted in Figure 1. It is intriguing that including the word “nano” in the search causes the bibliographic data to pass through a maximum value of around 2015. This indicates the achievement of some kind of technological excellence in the preparation of exchange-biased structures.

In this paper, we provide an update of the recent developments in the experimental and theoretical investigations of EB in nanostructures, published since 2005. The review is structured as follows: After a brief overview of the properties of exchange-biased nanostructures and exchange bias modeling approaches specifically aiming at nanostructures, a general introduction to the interplay of exchange bias and shape anisotropy is provided. In the subsequent sections, nanostructured exchange-biased systems are reviewed, starting with Co/CoO as one of the most often investigated material systems, followed by cobalt oxides coupled with other ferromagnets or ferrimagnets, in addition to systems containing NiO, FeO, and finally systems from other material combinations.

## 2. Properties of Exchange-Biased Nanostructures

**Figure 2:**
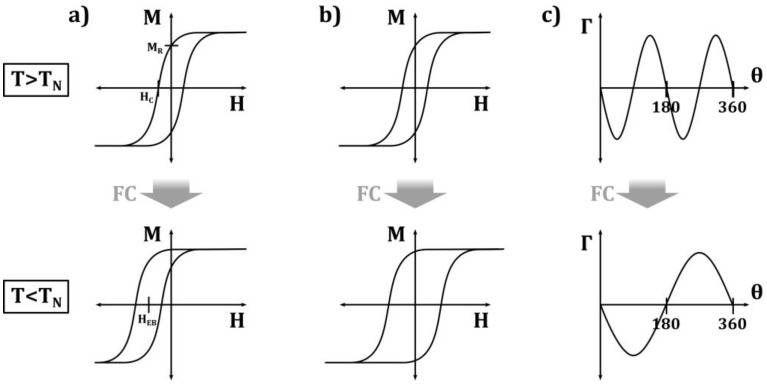
Schematic representation of the main effects induced by the FM–AFM exchange coupling causing (**a**) loop shift, (**b**) coercivity enhancement, and (**c**) unidirectional anisotropy. Redrawn illustration from [6].

Generally, exchange bias systems consisting of a ferromagnet and an antiferromagnet show a horizontal shift of their magnetization hysteresis loop when they are cooled through the Néel temperature of the AFM (Figure 2a). This is often accompanied by a broadening of the loop (Figure 2b), a vertical loop shift, or an asymmetry of the loop, which are attributed to unidirectional exchange bias anisotropy [6].

All these effects are temperature-dependent, with a larger EB typically at lower temperatures, while a sign change in the loop shift near the Néel temperature is also possible [15,16]. The blocking temperature *T_B_*, above which no EB is visible, can be significantly lower than the Néel temperature.

Other parameters affecting the exchange bias are the thickness of the layers or shells [5], the cooling field [17], the roughness of the interface between the FM and AFM [18], as well as the AFM orientation and crystallinity [5,19].

Especially in nanostructured EB systems, other effects may occur. Generally, a size reduction in a single FM or AFM may lead to a change in magnetic properties, as compared to bulk or even thin film materials [20,21,22,23,24]. The superposition of the EB-induced anisotropy with the shape anisotropy, which becomes increasingly important at smaller scales, can lead to even more interesting and partly counterintuitive results [25,26,27]. This is why this review focused on the EB in nanostructures, such as core–shell particles, nanodots, nanowires, nanorings, etc.

As the reader can recognize, there are no clear dependencies of the exchange bias on the material, structural, or other parameters—a thicker AFM can increase or decrease the EB field, a larger cooling field can increase or decrease the EB or even switch its sign, another crystal orientation can completely change the temperature-dependent asymmetry of the hysteresis loop, etc. This review thus cannot aim to conclude the most important facts, which are valid in all EB systems; however it aims to present a broad overview of the typical findings and unexpected results in the experimental and theoretical investigations of diverse exchange-biased nanostructures.

## 3. Modeling Exchange Bias in Nanostructures

While the naïve conception of EB as a frozen internal magnetic field is sufficient to explain the horizontal loop shift [28,29,30], the absolute value of the shift as well as the other effects observed in different exchange bias systems necessitate more sophisticated models. Most of them are based on thin film systems; nevertheless, they can be extended to other geometries.

**Figure 3:**
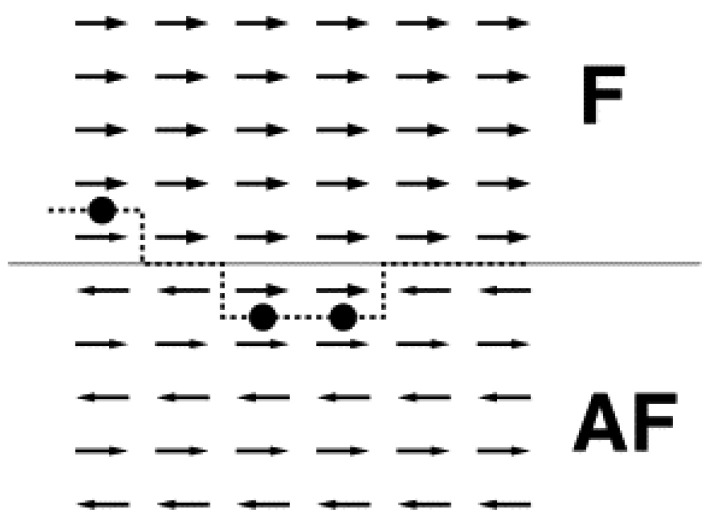
Ferromagnet (F)/antiferromagnet (AF) thin film system with antiferromagnetically coupled interface. The roughness (dashed line) results in frustrated interactions (dots). Reprinted from [31], Copyright 2001, with permission from Elsevier.

Early approaches added domain walls perpendicular to the interface, investigated the influence of the interface, and proceeded from a purely parallel/antiparallel orientation of the AFM and FM spins to canted or non-collinear spins [31]. Malozemoff firstly introduced domain walls perpendicular to the FM/AFM interface, frozen at low temperatures, so that the domain wall energy was added to the interface energy [32,33,34]. These models assumed a rough interface and a compensated spin structure, as shown in Figure 3 [31].

**Figure 4:**
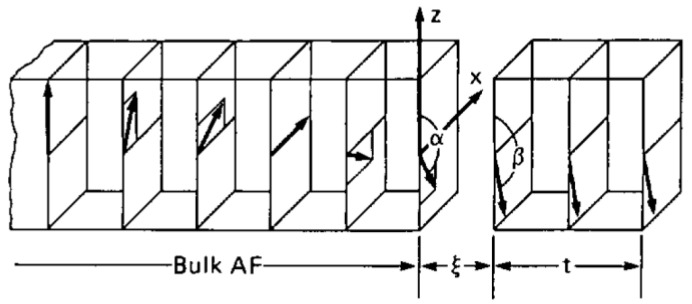
Magnetic model of a bulk AFM (only one sublattice’s spins are shown) in contact with a FM thin film. Here, the uniaxial AFM anisotropy is oriented along the positive *z*-direction, while the external magnetic field is oriented along the negative *z*-direction. Reprinted from [35], Copyright 2001, with the permission from AIP Publishing.

Non-collinear orientation of FM and AFM spins, as depicted in Figure 4, were introduced in Mauri’s model [35], resulting in the same equation for the EB field as the older Meiklejohn–Bean model [2].

A three-dimensional Heisenberg model describing a single-crystalline ferro- and antiferromagnets with antiferromagnetic coupling at the flat interface was suggested by Schulthess and Butler, showing that the FM spun oriented perpendicularly to the AFM easy axis [36,37]. By introducing the slight canting of the AFM spins, the so-called 90° coupling between the AFM and FM formed, resulting in an increased coercive field. While the flat surface in their model could not explain the exchange bias shift, introducing defects at the interface was found to also lead to an exchange bias in the model [38,39].

Combining a single-crystal ferromagnet with a polycrystalline AFM, a Heisenberg model was suggested by Stiles and McMichael [40]. Their results were similar to those of Mauri and, if they added a 90° coupling, showed a strong dependence on this parameter.

**Figure 5:**
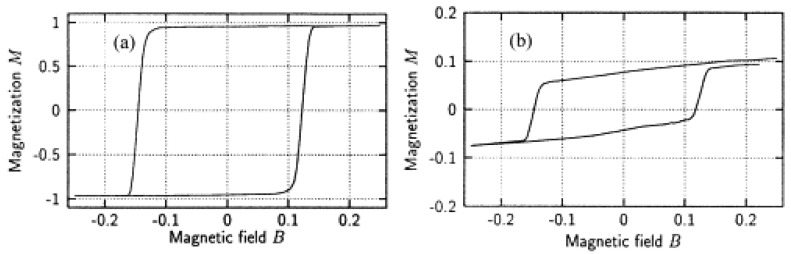
Hysteresis loops of (**a**) the ferromagnet; (**b**) the interface layer of the AFM. Reprinted from [45], Copyright 2001, with permission from Elsevier.

Switching from defects at the interface to defects in the AFM, the domain state model used a so-called diluted AFM [41,42,43,44], as depicted in Figure 5 [45]. Applying Monte Carlo simulations, it could not only model the exchange bias shift, but also the vertical shift of the hysteresis loop as well as the training effect.

A model, especially for Fe/FeF_2_ and Fe/MnF_2_ or similar systems with large AFM anisotropies, was developed by Kiwi et al. [46,47,48]. In such AFMs, the domain walls were much thinner than in the FM, leading to a perpendicular orientation of AFM and FM spins and spin canting in the interface AFM layer as well as incomplete FM domain walls [49,50]. These models calculated quantitatively realistic EB shifts for the aforementioned systems and even managed to model the positive EB, which was sometimes found here [48,49,50].

More recently, many new models were published, mostly focusing on specific material systems and the correlated effects accompanying the EB [51,52,53]. These models, however, are mostly based on thin film EB systems.

Nevertheless, there are also attempts specifically aiming at understanding and predicting nanostructured EB systems. For core–shell nanoparticles (NPs), Dimitriadis et al. developed an atomistic Heisenberg model with uniaxial anisotropy [54]. For spherical particles, i.e., the most often used ones, they found larger numerical exchange bias values than for cubical particles, due to the mostly compensated interfaces in the latter. Evans et al. used a classical atomistic spin model to describe the magnetic properties of core–shell FM–AFM nanoparticles with rough interfaces, finding a high degree of variation in the calculated EB fields for similar particles [55,56]. In a previous atomistic modeling of Co/CoO nanoparticles, the group observed the high thermal stability of the FM in the bias direction, suggesting that both FM and AFM should be switched in heat-assisted recording, leading to a new thermally stable state after cooling through the Néel temperature again [57].

A Monte Carlo simulation was used by Iglesias et al. to show that increasing the exchange coupling across the interface of a core–shell particle resulted in an increased EB shift and increased asymmetry of the hysteresis loop, since magnetization reversal occurred by different mechanisms in both branches of the loop [58]. The same group used a subsequent Monte Carlo simulation to model core–shell nanoparticles, resulting in the temperature-dependence of the horizontal loop shift, combined with a vertical loop shift, enlarged coercive fields, and even asymmetric hysteresis loops [59]. In this simulation, the EB field was found to be correlated with the net magnetization of the interface spins. Eftaxias and Trohidou found the same correlation, while in their Monte Carlo simulation of core–shell nanoparticles, the coercive field was found to mostly depend on the size of the interface [60]. Nanoparticles with a ferromagnetic core and ferrimagnetic shell were investigated in a Monte Carlo simulation by Vasilakaki and Trohidou who showed that a thicker shell increased the EB and reduced the remanent magnetization as well as vertical shift and training effect [61].

For an antiferromagnetic matrix with embedded ferromagnetic cores, Hu and Du used a simulation with a modified Monte Carlo simulation to show the possibility to switch the EB field from usual negative values to positive values with larger cooling fields in case of antiferromagnetic interface coupling, while for ferromagnetic interface coupling, the EB field was always negative and quantitatively not influenced by the cooling field [62].

In addition to atomistic and Monte Carlo simulations, some authors presented micromagnetic simulations of exchange-biased nanostructures. Heinonen et al. developed a micromagnetic simulation to investigate permalloy (Py) disks of 1 µm diameter and 12 nm thickness on an IrMn antiferromagnet with a grain size of 25 nm [63]. They observed higher gyrotropic and spin-wave eigenmode frequencies of the vortex oscillations in the Py disk with a higher EB, thus allowing tailoring the magnetization dynamics via the EB. They also observed a polarity change in the vortex core, which they assumed to be correlated with the grain structure of the AFM.

Li et al. used a micromagnetic solver OOMMF to simulate ferromagnetic iron dots with of diameters 300 and 600 nm and a thickness of 30 nm with and without coupled AFMs [64]. They reported the strong influence of shape anisotropy on the smaller dots, resulting in a flux-closed vortex state for the pure iron dots and identical magnetization reversal processes for the biased dots.

However, there are also simpler models found in the literature, usually based on Stoner–Wohlfarth models, which allow for quicker, but naturally less accurate, simulations of nanoscale EB systems [65,66].

Finally, it should be mentioned that none of these models was capable of explaining all EB systems either qualitatively or quantitatively. This is why several different models still exist, each of which is useful to explain special EB systems with their specific properties.

## 4. Exchange Bias and Electronic Structure

Many researchers mention the influence of the electronic interface structure on the measured exchange bias.

Hirai et al. investigated Co/CoO_x_ layer systems where they applied an electric field strongly modulating the exchange bias perpendicular to the surface [67]. This finding was attributed to the modulation of the electronic interface state.

Vaz et al. studied the exchange bias and interface electronic structure in Ni/Co_3_O_4_ thin films [68]. Measuring photoelectron spectroscopy, they observed strongly varying oxidation states of Ni and Co_3_O_4_ at the interface and concluded that the exchange bias mostly resulted from a CoO interface layer of approximately 4 Å thickness combined with a monolayer of NiO.

X-ray absorption spectroscopy was used to identify electronic structures in a single LaMnO_3-δ_ film, revealing Mn^2+^ components with double exchange between Mn^2+^-O-Mn^3+^ in the upper (FM) part of the film, while the lower (AFM) part showed mostly Mn^3+^ [69]. This electronic phase separation perpendicular to the layer resulted in the measured exchange bias.

In another single-film EB system, CoFe_2_O_4_(111) grown on an Al_2_O_3_(0001) substrate, Yang et al. observed a large exchange bias, which they attributed to an AFM interface layer from CoO, as concluded from the electronic structure of the interface layer, coupling to the residual ferrimagnetic layer [70].

In an NiFe_2_O_4_ film, Jaffari et al. showed that modifying the electronic structure enabled a variation in the cationic distribution, resulting in the possibility to tailor the coercive field and to create an exchange bias due to random oxygen vacancies, which led to random anisotropy in the exchange-coupled grains [71].

Many other research groups investigated the correlation of the electronic structure at an interface or within a single film with an electronic structure gradient. However, this topic is only scarcely discussed for nanostructures [72,73] and thus not further discussed here.

## 5. Exchange Bias and Shape Anisotropy

The interplay between EB and shape anisotropy has been investigated by many research groups. While, most often, nanostructures are investigated, even microstructures, such as micrometer-scale stripes [74,75], dots [76], or similar structures [77], show recognizable effects of the structure on the overall magnetic sample properties. The most important is the shape anisotropy in the case of EB nanostructures.

For Co/CoO core–shell nanowires, Tripathy et al. found a strong EB dependence on the cooling field orientation with respect to the nanowire orientation [78]. Interestingly, they observed the exchange bias field, i.e., the horizontal shift of the hysteresis loop, to be smaller or larger than a continuous film of the same material composition, while the coercive field was always larger for the nanowire array, as can be expected since the strong shape anisotropy blocks the magnetization reversal.

Gandha et al. reported a large exchange bias for aligned Co/CoO core–shell nanowire systems with an unclear angular correlation between the measurement angle and EB, as well as the coercive field [79]. Maurer et al. studied the superparamagnetic fluctuations in the antiferromagnetic shell in Co/CoO nanowires, which they described to be specifically found in 1D systems due to their large shape anisotropy [80]. In a more complex setup, Koplak and Morgunov measured the position-dependent EB and coercivity of α-Fe/PrDyCoFeB core/shell microwires, and found the shape anisotropy to be responsible for multi-domain states in shorter and single-domain states in longer microwires [81].

For a quasi-two-dimensional periodically nanostructured Co filament, which was naturally oxidized, Huang observed magnetoresistance, which he attributed to the competition between the local shape anisotropy and dipolar interaction of the periodic nanostructures, as well as the exchange coupling between the Co core and CoO shell [82]. For Co/BiFeO_3_ core–shell nanostructures, Ali et al. mentioned the role of the strong shape anisotropy to define the easy magnetization axis, in this way also defining the angular-dependence of the EB [83].

**Figure 6:**
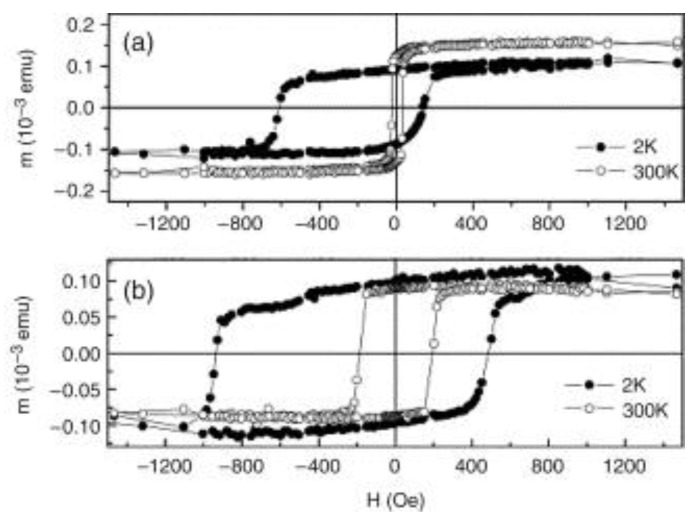
Hysteresis loops for oxidized Co samples with the same thickness of 40 nm: (**a**) continuous film; (**b**) patterned film with stripes. Reprinted with permission from [85], Copyright 2007 Elsevier.

Zhang et al. used nanoimprint lithography to prepare metallic wire arrays from Co/IrMn thin film systems and observed the angular dependence of the magnetization to be dependent on the ratio of the shape anisotropy and EB anisotropy [84]. Similarly, Rosa et al. produced oxidized Co line arrays by interference lithography and observed the strong difference of the hysteresis loop, as compared to a Co thin film, as depicted in Figure 6 [85]. The authors ascribed the broadening of the hysteresis curves to the shape anisotropy, strongly favoring magnetization orientation along the line structure orientation. On the other hand, they observed the reduction in remanence at low temperatures for the continuous film (Figure 6a), which they attributed to thermal stress due to different thermal expansion coefficients of the Co layer and the substrate was not visible for the stripe sample where such stress was negligible.

While the shape anisotropy is especially important in one-dimensional structures, it can also play an important role in two-dimensional patterned EB structures. Investigating elongated Co/CoO nanorings, Tripathy et al. reported a competition between the shape anisotropy favoring the major ring axis and the unidirectional anisotropy whose direction is defined by the cooling field orientation, resulting in significant changes in the EB field with the measurement and cooling field angle [86]. Eisenmenger et al. prepared square arrays with circular dots from an Fe/FeF_2_ film and found that both branches of the hysteresis loop of this EB system were affected differently by the shape anisotropy, resulting in a strong anisotropy especially visible in the transverse magnetization [87].

In triangular, pentagonal, and heptagonal exchange-biased permalloy nanodisks, Gong et al. observed the largest EB necessary to control the vorticity of the magnetic vortex formed at a 500 nm diameter, which was identical to the diameter where the shape anisotropy presented the greatest contribution to the vorticity control [88]. The stabilization of vortices by shape anisotropy was also suggested by Albisetti who investigated nanostructured CoFeB/IrMn/Ru squares [89]. Moralejo et al. improved the stability of magnetization reversal by a vortex state by tailoring the combination of EB, shape anisotropy, and inter-element spacing in arrays of elliptical NiFe/IrMn EB elements [90]. More complex structures, in which the combination of EB and shape anisotropy was used to tailor magnetization reversal by changing the system’s dimensions, contain planar Hall effect sensor crosses [91], L-shaped [92], or zigzag exchanged-biased nanostructures [93].

The elemental composition of nanoparticles has, apart from their shape, the strongest influence on the magnetic properties of the systems. The following sections thus discuss several EB systems that were studied as nanostructures.

## 6. Co/CoO Nanostructures

Co/CoO is still the most often used material combination in exchange bias systems, regarding thin films as well as nanostructures or core–shell particles [7,94,95]. Amongst the potential shapes of such Co/CoO nanostructures, core–shell nanoparticles are still the easiest to produce. Nogués et al. produced pseudo-multilayers of NPs with an average core diameter of 4 mm and average shell thickness of 1 mm in an Al_2_O_3_ matrix, testing different coverage densities of the NPs from 8–33% [96]. They observed an increase in the EB field by a factor of 400 with an increasing coverage density, which they attributed to the important role of the interacting shells to stabilize NP magnetism. In a similar setup, Dobrynin et al. investigated the influence of the nanoparticle diameter and showed that nanoparticles with a 2–3 nm diameter were generally too small to show a horizontal EB shift, while they showed enlarged coercivity and a vertical shift of the hysteresis loop [97].

Inderhees et al. investigated core–shell Co/CoO particles with different degrees of oxidation [98]. They reported an impact of oxidation on the decompensation of the core–shell interface, resulting in a large exchange bias due to the highly ordered interface and enhanced core–shell coupling by uncompensated interface moments. Using radio-frequency transverse susceptibility measurements, Chandra et al. showed that the hysteresis loops measured below the freezing temperature of the CoO shell always showed asymmetry, while no asymmetry was visible above this temperature; although, the blocking temperature, i.e., the temperature below which an EB shift occurs, was higher than the shell freezing temperature [99]. Comparing core–shell nanoparticles with diameters of 11 nm with different shell thicknesses, Feygenson et al. observed a maximum EB shift for a shell thickness of approximately 1 nm, while the coercive fields were largest for a thickness of approximately 2–3 nm [100].

Extending core–shell nanoparticles along one axis leads to core–shell nanorods or nanowires. Proenca et al. used electrodeposition to grow Co nanowires and nanotubes in nanoporous alumina templates and let the inner walls oxidize towards CoO [101]. The exchange bias showed a blocking temperature of around 220 K and a doubled exchange bias shift for measurements perpendicular to the tube long axis, as compared to the measurements parallel to this axis.

**Figure 7:**
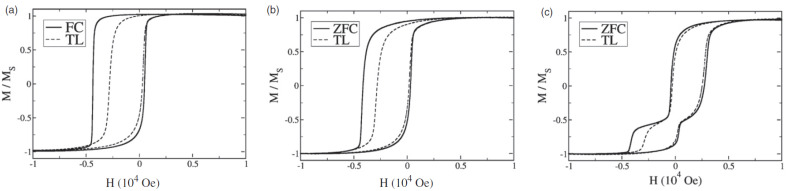
Magnetic hysteresis loops of Co clusters in a thin CoO matrix, obtained at 5 K after cooling down from 300 K. (**a**) FC at 10 kOe, training loop (TL); (**b**) ZFC after saturation in a positive field and training loop; (**c**) ZFC from a partially demagnetized state, and training loop. From [102], originally published under a CC-BY license.

Another structure of an exchange-biased Co/CoO system was reported by Dobrynin et al. who embedded Co clusters with a diameter of 2 nm in a thin CoO matrix [102]. They found a doubled hysteresis loop, as depicted in Figure 7c, which they attributed to the superposition of exchange spring and exchange bias effects in this system, where the exchange spring effect describes aligning FM clusters in an AFM matrix. Moreover, all measurements after field cooling (FC) and zero-field cooling (ZFC) were repeated and showed a strong training effect, i.e., a significant change in especially the left branch of the hysteresis loop (Figure 7a,b). In a similar way, de Toro et al. produced Co/CoO nanocomposites by sputtering Co in an oxygen atmosphere, resulting in Co nanoparticles embedded in a CoO matrix, which showed an EB at low temperatures, while no double loops were reported [103].

Another nanostructured film was prepared by Tripathy and Adeyeye in the form of an antidot array with a thin Co layer of 25 nm thickness at the bottom, followed by a 5 nm CoO [104]. For low temperatures, they found the exchange bias approximately doubled, as compared to the continuous thin film system, while the blocking temperature was near 270 K for both systems. Similarly, the coercive fields more than doubled for the whole temperature range up to 300 K. Additionally, using photolithography, Luo and Misra prepared lattices with diamond- or triangular-shaped nanostructures as dots or antidots [105]. They found an exchange bias at low temperatures and a significantly increased coercivity, which they attributed to the shape anisotropy in the nanostructures.

Kovylina et al. produced granular thin films from Co nanoparticles with varying degrees of oxidation inside a zirconia matrix by the pulsed laser ablation of a Co/zirconia target in a chamber with varying O_2_ pressure [106]. Both the coercive field and exchange bias depended on the degree of oxidation, with 10^−3^ mbar resulting in a maxima of both values, while they were minimized at much higher or much lower oxygen pressures, when the nanoparticles were nearly fully oxidized or purely metallic Co.

While Co/CoO is a well-studied EB system, as thin films or in the form of nanostructures, cobalt oxide can also be combined with other materials to form exchange bias nanosystems, as is discussed in the following section.

## 7. Other Exchange-Biased Nanostructures Containing Cobalt Oxides

**Figure 8:**
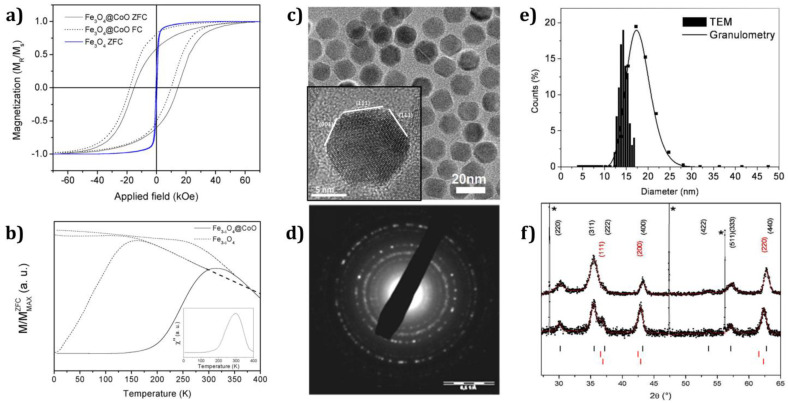
(**a**) Comparison of the magnetic properties of Fe_3−δ_O_4_@CoO and Fe_3−δ_O_4_ nanoparticles by measuring *M*(*H*) curves at 5 K; (**b**) ZFC and FC magnetizations versus temperature curves and imaginary part of the susceptibility *χ*″ measured under a 3.5 Oe alternative field at 1 Hz for Fe_3−δ_O_4_@CoO NPs (inset); (**c**) transmission electron microscopy (TEM) micrograph of Fe_3−δ_O_4_@CoO NPs; (**d**) electronic diffraction pattern; (**e**) size distribution measured from TEM micrographs and hydrodynamic diameter measured by granulometry; (**f**) X-ray diffraction patterns (black) of Fe_3−δ_O_4_ (up) and Fe_3−δ_O_4_@CoO core−shell (down) nanoparticles and profile-matching refinement (red). Peaks are indexed to *hkl* reflections of Fe_3−δ_O_4_ (up in black) and CoO (down in red). Stars show peaks corresponding to silicone, which is used as an internal standard. Adapted with permission from [107], Copyright 2013, American Chemical Society.

Fe_3−δ_O_4_@CoO core–shell nanoparticles with a 2 nm CoO shell around the Fe_3−δ_O_4_ core (11 nm diameter; Figure 8e) were grown by Baaziz et al. (Figure 8) [107]. The characterization of these nanoparticles revealed the epitaxial growth of CoO on the maghemite surface of the Fe_3−δ_O_4_ nanoparticles (Figure 8c), resulting in a high-quality interface. They observed a large exchange bias field of more than 4 kOe and a large coercive field of 15 kOe, which was significantly increased as compared to pure Fe_3−δ_O_4_ nanoparticles, as depicted in Figure 8a. The blocking temperature was observed to be 293 K (Figure 8b), i.e., identical to the bulk Néel temperature of CoO [107].

Panagiotopoulos et al. investigated γ-Fe_2_O_3_/CoO and CoO/γ-Fe_2_O_3_ core/shell particles and observed quite different temperature dependencies for both types of exchange-biased particles, where the ferrimagnet as core showed an exchange bias up to around 200 K, while the AFM as core resulted in a blocking temperature close to 60 K [108]. Similarly, Lavorato et al. prepared nanoparticles with a CoO core and Co_1−x_Zn_x_Fe_2_O_4_ ferrimagnetic shell and observed that the EB was the maximum for x = 0.25, while it vanished for samples without Zn [109].

CoPt/CoO nanocomposites as well as CoO nanoparticles were investigated by Tomou et al. [110]. While CoPt nanoparticles with CoO shells showed an EB below 20 K, a clear horizontal shift of the hysteresis loop was also observed for pure CoO nanoparticles. This finding was attributed to weak ferromagnetism due to the uncompensated surface spin of the antiferromagnetic particle, an effect which is well-known for nanoparticles [111].

Similarly, Salabas et al. produced Co_3_O_4_ nanowires with an average diameter of 8 nm by nanocasting in a nanoporous silica matrix [112]. They found that the surface spins showed a spin glass behavior, resulting in a clear EB shift, enlarged coercive field, and vertical shift of the hysteresis loop below the Néel temperature of the AFM near 30 K. The authors explained the surface spins to be partly frozen-in, resulting in the EB, while the other surface spins behaved similar to a ferromagnet and were aligned by the external magnetic field. They also mentioned that only a slow anisotropy variation throughout the AFM core could result in the observed vertical shift and broadened hysteresis loop, while a sharp border between the core and shell should not result in an enhanced coercivity [112]. Similarly, Wang et al. found a large exchange bias shift in Co_3_O_4_ nanorods produced by hydrothermal synthesis for measurements below the Néel temperature [113].

Another way to work with Co_3_O_4_ surface spins was reported by Dutta et al. who compared microparticular (average diameter: 1–2 µm) and nanocrystalline Co_3_O_4_ (average diameter of 17 nm) regarding their magnetic properties [114]. They found a Néel temperature of 30 K for microparticular and 26 K for nanocrystalline Co_3_O_4_ particles, both lower than the bulk value of 40 K. While a closed hysteresis loop without a horizontal shift was found for the microparticular Co_3_O_4_, a temperature-dependent broad coercivity and exchange bias shift were visible for the nanoparticles below 26 K.

## 8. Ni/NiO Nanostructures

Similar to Co/CoO, Ni/NiO can also be found in the form of core/shell particles. Querejeta-Fernández et al. described the preparation of such nanoparticles with an average diameter of 10 nm by the thermal decomposition of a medium containing a Ni^2+^ salt, followed by a reduction step to yield Ni crystallization and finally the oxidation of the shell [115]. They observed large EB shifts for small- and middle-core diameters and smaller EB fields for larger cores with thin NiO shells. Johnston-Peck et al. used solution chemistry with subsequent solution-phase oxidation instead of preparing Ni/NiO core–shell nanoparticles with shell thicknesses of 2–3 nm and core diameters of 8–24 nm [116]. While the temperature-dependence of the sample magnetization depended on the core and shell diameters, these samples generally showed no horizontal EB shifts, but small increases in coercivity indicating a weak EB. For core–shell particles prepared by a sol-gel route with diameters of 8–27 nm, Thakur et al. investigated the cooling field dependence and observed a slightly reduced EB for cooling fields larger than 20 kOe [117].

Rinaldi-Montes et al. prepared Ni/NiO core/shell nanoparticles by the pyrolysis of an inorganic precursor in the pores of an active carbon matrix, followed by oxidation in air [118]. These nanoparticles showed a shell thickness of 2 nm and varying core diameters, depending on the pyrolysis temperature. The authors reported that the shell froze into a spin glass state below approximately 40 K, correlated to an EB shift of the measured hysteresis loops below this temperature, which was far below the bulk Néel temperature of NiO of 523 K.

While most studies of Ni/NiO nanostructures are based on core/shell structures, a few other exchange-biased Ni/NiO nanostructures were investigated. Kremenovic et al. prepared nanocomposites of 62% NiO with crystallite sizes of about 11 nm and much larger crystallite sizes of 278 nm for Ni [119]. Using thermal annealing in air, the NiO content and crystallite sizes increased, while the Ni crystallite sizes decreased. However, high-energy ball milling resulted in a reduction in the NiO content and overall decreased the crystallite size. An EB was found in milled samples with particle sizes of 10 nm for NiO and 11 nm for Ni, while larger crystallites resulted in a reduced coupling area and correspondingly vanishing EB.

## 9. Other Exchange-Biased Nanostructures Containing Nickel Oxides

**Figure 9:**
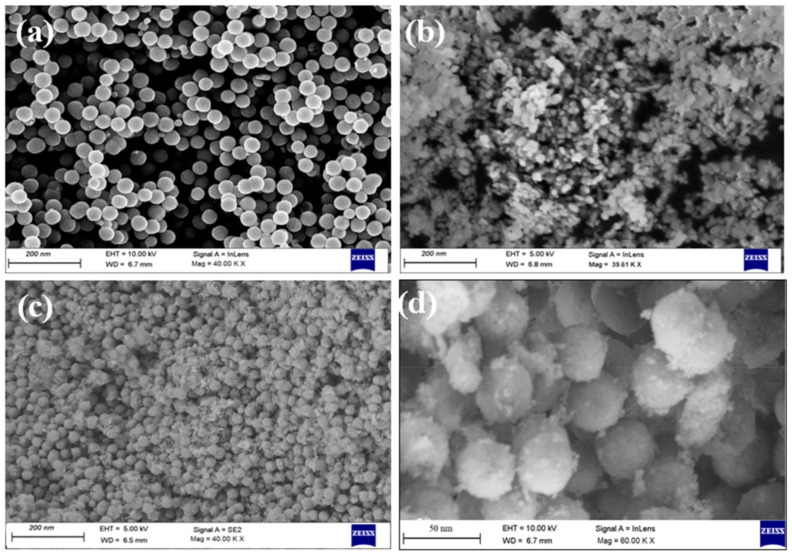
Scanning electron microscope images of (**a**) pure Fe_3_O_4_ and (**b**) NiO nanoparticles, as well as core–shell nanoparticles of (**c**) Fe_3_O_4_@NiO and (**d**) NiO@Fe_3_O_4_. Adapted from [120], originally published under a CC-BY license.

Similar to cobalt oxide nanostructures exchange-coupled to ferromagnets other than cobalt, there are also few reports about NiO combined with other ferro- or ferrimagnets. Tsopoe et al. prepared core–shell nanoparticles combining NiO with the ferrimagnet Fe_3_O_4_, testing the AFM as a core and shell, respectively [120]. They observed rod-shaped NiO nanoparticles, while pure Fe_3_O_4_ nanoparticles and both sorts of core–shell nanoparticles were spherical, as shown in Figure 9. All diameters were in the range of 30–50 nm. For both sorts of core–shell nanoparticles, the blocking temperature was around 200–250 K, with the highest EB of 330 Oe at 60 K observed for NiO@Fe_3_O_4_ core–shell nanoparticles. Interestingly, the authors observed an EB shift along the positive *x*-axis, i.e., opposite to the common direction, for Fe_3_O_4_@NiO core–shell particles, which they explained by more pinning of down-spins at the core–shell interface. The coercive fields of all nanoparticles, both pure and core–shell, decreased with the increasing temperature. Embedding NiFe_2_O_4_ ferrimagnetic nanoparticles in a NiO matrix, Tian et al. also observed a blocking temperature of approximately 250 K [121]. The authors explained the EB by the exchange interaction between the ferrimagnetic nanoparticles and the spin glass-like interface phase.

Such a spin glass state, formed below 10 K, was also mentioned by Rinaldi-Montes et al. who prepared NiO nanoparticles [122]. Similar to the aforementioned CoO or Co_3_O_4_ nanoparticles, they observed an EB for nanoparticles larger than a 2 nm diameter, which they attributed to the magnetic coupling between the AFM core and spin glass shell. Winkler et al. reported the spin glass state of 3 nm NiO nanoparticles to occur below 15 K [123]. Makhlouf et al. investigated the temperature dependence of the EB in NiO nanoparticles depending on the NP diameter and observed a lower blocking temperature and also smaller exchange bias shift for smaller nanoparticles, while the greatest EB was achieved for a nanoparticle diameter of 26 nm [124].

## 10. FeO-Based Exchange-Biased Nanostructures

While exchange-biased thin film systems with Fe as a ferromagnet often contain FeF_2_ or MnF_2_ as an antiferromagnet due to their interesting magnetic anisotropies [10,12], only very few nanostructures are based on these AFMs [87,125,126]. Most often, Fe/FeO and other nanostructures containing FeO are investigated instead.

Martínez-Boubeta et al. investigated naturally oxidized Fe nanoparticles with diameter of 5–13 nm, which were prepared by the thermal decomposition of iron pentacarbonyl, followed by oxidation in air [127]. They observed low blocking temperatures of only 19 K for the core–shell nanoparticles with a diameter of 5 nm, while the largest NPs showed a blocking temperature of 160 K and greater EB shifts for larger particles. This finding is similar to the results of Makhlouf et al. who also recognized lower blocking temperatures and a smaller EB for smaller NiO nanoparticles [124]. Similarly, Unni et al. prepared single-crystalline Fe nanoparticles, which showed an EB after oxidation, while the addition of oxygen during the thermal decomposition synthesis resulted in pure magnetite nanoparticles [128].

In addition to antiferromagnetic FeO, there are other common iron oxides, e.g., ferrimagnetic magnetite (Fe_3_O_4_), ferrimagnetic maghemite (γ-Fe_2_O_3_), and antiferromagnetic hematite (α-Fe_2_O_3_) [129]. Especially Fe_3_O_4_/FeO is often investigated. Sun et al. prepared FeO/Fe_3_O_4_ core/shell nanoparticles by oxidizing FeO nanoparticles at different temperatures and observed a large exchange bias shift with clear loop asymmetry, both of which depended on the relative dimensions of the core and shell [130]. Nanocomposite Fe_3_O_4_/FeO nanoparticles were prepared by pulsed laser irradiation in ethyl acetate and showed a positive correlation of the coercive field and EB with the relative fraction of FeO, as well as a blocking temperature close to the FeO Néel temperature of 198 K [131].

## 11. Other Iron-Oxide-Based Exchange-Biased Nanostructures

Similar to CoO, Co_3_O_4_, and NiO, iron oxides can also form EB systems from single-phase materials due to the interaction between the core and surface spins. Shevchenko et al. reported growing gold/iron oxide core/hollow shell nanoparticles with coexisting γ-Fe_2_O_3_ and Fe_3_O_4_ phases, i.e., both ferrimagnetic materials [132]. They attributed the large EB shift observed at temperatures below approximately 50 K to a spin glass layer forming on the nanoparticle surface. A similar effect was also reported by Chandra et al. who attached one or many Fe_3_O_4_ nanoparticles to Au seed particles [133]. They found an EB as long as the clustered growth resulted in highly disordered Fe_3_O_4_ surface spins, while the EB vanished with the vanishing interfacial stress. In contrast, investigating Au-Fe_3_O_4_ dumbbell nanoparticles, Feygenson et al. identified an antiferromagnetic FeO phase at the interface between Au and Fe_3_O_4_ as the reason for the EB shift [134].

An interesting material combination was chosen by Chandra et al. who grew Fe/γ-Fe_2_O_3_ core–shell nanoparticles, i.e., combining a ferromagnetic core with a ferrimagnetic shell [135]. They observed a large EB below approximately 35 K, when the ferromagnetic core was frozen and the ferrimagnetic shell moments started blocking, of up to 3 kOe, with slightly higher values for a cooling field of 2 T than for a cooling field of 5 T. A systematic study of this system revealed that below a particle diameter of approximately 10 nm, the surface spins were mainly responsible for the EB, while for larger particles, the interface between Fe and γ-Fe_2_O_3_ was the dominant contribution [136].

Comparing core–shell particles with MnFe_2_O_4_ (soft ferrite) or CoFe_2_O_4_ (hard ferrite) cores and a spin glass-like *γ*-Fe_2_O_3_ shell, Cabreira-Gomes et al. found an EB, which was strongly related to the cooling field [137]. The authors reported a “supershell matrix” forming when the nanoparticle shells made contact, further increasing the EB shift.

Maltoni et al. compared nanoparticles from γ-Fe_2_O_3_ with Co-doped γ-Fe_2_O_3_ nanoparticles and observed an EB only in the latter, as well as in the mixture of both sorts of nanoparticles [138]. In polycrystalline hollow γ-Fe_2_O_3_ nanoparticles of 9 and 19 nm, Khurshid et al. observed an EB in the larger nanoparticles due to inner and outer surface spin disorders, but attributed the loop shift and open hysteresis loop of the smaller ones to measuring minor loops, even in external magnetic fields of 9 T [139]. This aspect of minor loops as a pseudo-EB is discussed in the final section of this paper.

**Figure 10:**
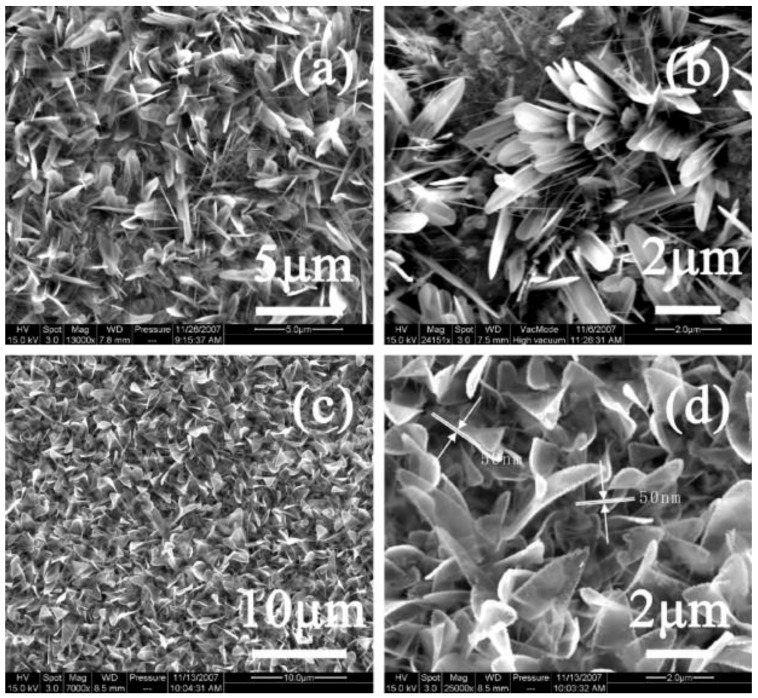
SEM images of two typical kinds of nanoleaves: (**a**,**b**) sword-like nanoleaves; (**c**,**d**) board nanoleaves. Reprinted with permission from [141], Copyright 2009, Elsevier.

There are also reports of EB in α-Fe_2_O_3_-based nanoparticles. Bhowmik and Saravanan observed an EB in α-Fe_2_O_3_ nanograins, which they attributed to their core–shell spin structures, with the core having the bulk magnetic properties and the shell being influenced by the surface modification upon the mechanical milling of the sample [140]. Despite round nanoparticles, Xu et al. suggested α-Fe_2_O_3_ nanoleaves, prepared by the oxidation of pure iron, as depicted in Figure 10 [141]. They found a blocking temperature of 120 K and a small EB after field cooling at 2 T, which they attributed to the surface magnetization being different from the magnetization in the core or a small amount of Fe_3_O_4_ being formed during oxidation.

Fe_3_O_4_ can also be used in core–shell nanoparticles in combination with another magnetic material. Ong et al. investigated a monodisperse Fe/Fe_3_O_4_ core/shell as well as Fe_3_O_4_ hollow-shell nanoparticles and observed a much larger EB in the first, but sharp demagnetization jumps at low fields due to the sudden switching of shell magnetic moments for both nanoparticles [142]. Nunez et al. recently reported a large coercivity of nearly 6 kOe at 5 K and an EB for Fe_3_O_4_/MgO/CoFe_2_O_4_ core–shell-shell nanoparticles, which they attributed to the freezing of the surface spins, pinning the magnetic moments of the CoFe_2_O_4_ shell [143]. Combining Fe_3_O_4_ with manganese oxide as the core or shell, Estrader et al. observed a horizontal EB shift in three different nanostructures after cooling in small fields, while a large cooling field resulted in a sign change in the EB for both structures with iron oxide cores and reduced the EB to nearly zero for the nanoparticles with manganese oxide cores [144].

An EB shift was also observed in Zn_1−*x*_Fe*_x_*O (*x* ≤ 0.08), growing in a wurtzite structure and showing ferromagnetism at room temperature [145]. For this system, the EB shift depended on the Fe concentration, while the origin of this EB shift was not investigated further.

## 12. Other Exchange-Biased Nanostructures

The previous sections described the most common ferromagnets and antiferromagnets used in exchange-biased nanostructures. Many other EB systems, however, contain alloys of these common materials or are built from other materials, which are discussed in this section.

### 12.1. Exchange-Biased Nanostructures Containing Fe

Many EB systems contain Fe in the ferro-/ferri- or antiferromagnets. Amongst the antiferromagnets containing iron, FeF_2_ was previously mentioned. For Ni/FeF_2_, Basaran et al. showed that defects in the AFM bulk, produced by He-ion bombardment, significantly modified the EB shift [146]. Rodríguez et al. showed the possibility to manipulate the AFM spin structure and the resulting EB in Ni/FeF_2_ bilayers patterned with antidots [147].

**Figure 11:**
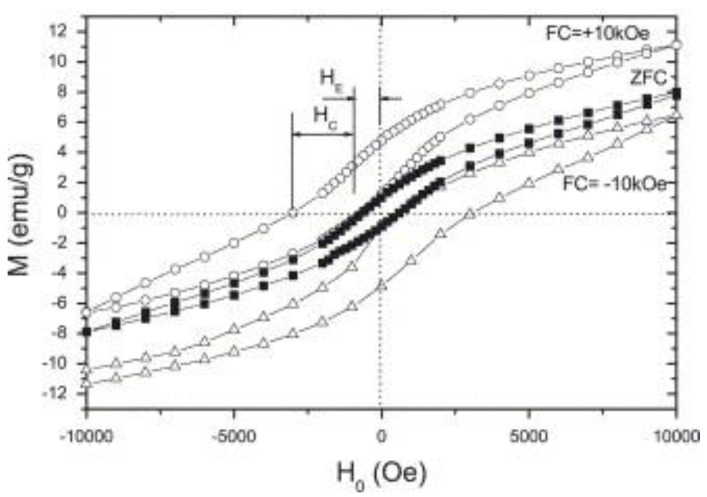
ZFC and 10 kOe FC magnetic hysteresis loops obtained at 4.2 K for the sample 20(Fe):80(MnO_2_) milled for 100 h. The open circles correspond to the +10 kOe FC experiment, while open triangles correspond to the −10 kOe FC experiment. The ZFC experiment is represented by the closed squares. Reprinted with permission from [148], Copyright 2006, Elsevier.

Fe, as the ferromagnetic part of an EB system, on the other hand, can be combined with different antiferromagnets. In milled nanostructured Fe/MnO_2_ with different Fe:MnO_2_ ratios, Passamani et al. not only found a horizontal EB shift, but also a strong vertical shift for the sample consisting of 20% Fe and 80% MnO_2_, as depicted in Figure 11, for positive and negative cooling fields [148]. In Fe/Cr core–shell nanoparticles with a core diameter of 2.7 nm, Binns et al. reported exchange bias to occur if a minimum of 2 atomic Cr layers surrounded the Fe core, in this way stabilizing the FM/AFM interface [149].

More often, however, Fe alloys are used in EB systems. Mumtaz et al. reported an EB in cobalt ferrite nanoparticles with diameters of 15–48 nm as well as a vertical shift and attributed the EB to the interface between the surface and ferrimagnetic core [150]. The same group prepared magnetic nanoparticles from Ni-doped cobalt ferrite (Co_1−*x*_Ni*_x_*Fe_2_O_4_) and observed a decreasing exchange bias and vertical shift for reduced Ni concentrations and a decreasing blocking temperature with increasing amount of Ni [151]. Core–shell nanoparticles with a cobalt ferrite core and CoFe_2_ shell showed exchange-spring coupling for thick shells of 8 nm or larger and an EB strongly decreasing with an increasing shell thickness [152]. Nanoparticles with a cobalt ferrite core and MnO shell showed an EB due to the ferrimagnetic/antiferromagnetic interface [153]. Co-doped FeO (Co_0.33_Fe_0.67_O) nanoparticles with a cobalt ferrite shell showed a large EB shift as well as an increased coercive field and a clear vertical shift for temperatures up to approximately 200 K, i.e., above the Néel temperature of pure FeO [154]. In IrMn/CoFe nanostructures, a maximum EB was observed for diameters of around 28 nm, approximately twice as large as in the corresponding continuous thin films, which was attributed to the reduction in FM and AFM domain sizes [155].

In Zn-substituted nickel ferrite (Zn_0.3_Ni_0.7_Fe_2_O_4_) nanoparticles with diameters of 5–33 nm, an EB shift of 1.2 kOe was observed, which was quite large for a single-phase system and was attributed to the interaction between disordered surface spins and highly ordered core spins [156]. NdFeO_3_ nanoparticle systems also showed an EB at low temperatures with only a weak training effect, which was not investigated further [157]. Pure nickel ferrite was used as a shell around an antiferromagnetic BiFeO_3_ core and not only caused an EB, as expected due to the exchange coupling at the interface, but also showed reduced coercivity after field cooling, as compared to zero-field cooling, which was the opposite to the usual behavior of EB systems [158].

Spizzo et al. investigated the transition from thin film to dot arrays for IrMn/NiFe nanostructures [159]. They observed decreasing exchange bias at 300 K upon reduced dot sizes, but an increasing EB shift at 10 K for reduced dot sizes. This was attributed to a structurally disordered, spin glass-like IrMn interface layer, which froze below 100 K into a stabilized regime. A positive exchange bias was reported for nanostructured FeMn/Co/FeMn networks prepared in a porous template [160]. While the thin film from the same material system showed a slightly decreasing EB with the increasing temperature, as expected, the nanostructured network exhibited a change in the sign of the EB close to 200 K, in addition to a significantly larger coercive field as compared to the thin film system.

Nanoparticles from antiferromagnetic LaFeO_3_ were investigated by Ahmadvand et al. who observed an EB, which they attributed to the exchange coupling between the core and shell showing weak ferromagnetism [161]. Similarly, BiFe_0.8_Mn_0.2_O_3_ nanoparticles, consisting of an AFM core and a diluted antiferromagnetic shell, showed an EB due to the interface exchange coupling between the core and shell [162].

Several studies were based on bismuth ferrite (BiFeO_3_), which is a canted antiferromagnet in bulk form. Single-crystalline BiFeO_3_ nanoparticles showed an EB, which was attributed to the interaction between the ferromagnetic surface and antiferromagnetic core spins [163,164]. In addition, Mazumder et al. reported the coupling of ferromagnetic and ferroelectric order parameters in nanoscale BiFeO_3_ [165]. In BiFeO_3_-CuO nanocomposites with ferromagnetic BiFeO_3_ nanocrystals with a diameter of 9 nm, embedded in antiferromagnetic CuO with different ratios, Chakrabarti et al. observed a strong EB below 170 K, with a maximum at equal amounts of BiFeO_3_ and CuO, due to blocked spins along the interfaces and strong magnetic exchange coupling between the FM and AFM [166]. In a nanocomposite of BiFe_3_O_3_ (~94%) and Bi_2_Fe_4_O_9_ (~6%), Maity et al. even found a spontaneous EB after zero-field cooling up to 300 K [167]. They reported a strong effect of the maximum external magnetic field applied and its sign on the measured EB, with the maximum EB achieved at around 150 K. These effects were attributed to the interaction between the ferromagnetic Bi_2_Fe_4_O_9_ cores and the canted AFM spins in the surrounding matrix.

### 12.2. Exchange-Biased Nanostructures Containing Mn

In addition to iron, manganese is often found in EB systems. Mn can be combined with typical magnetic materials, e.g., to form ferrimagnetic CoMn_2_O_4_ nanoparticles, in which both spontaneous exchange bias and a conventional EB field of 3.3 kOe at 50 K were observed [168].

There are, however, other diverse Mn-based magnetic materials used in EB systems. Ir_x_Mn_1−x_ is a high-temperature antiferromagnet with a bulk Néel temperature of around 690–1000 K [169,170]. In sub-100 nm structures of Py/Ir_20_Mn_80_, Baltz et al. observed the strong dependence of coercivity and EB on the AFM layer thickness, with the EB shift becoming smaller or larger than the value for thin films for varying AFM thicknesses [171]. The same group observed strong thermal activation effects in this system, resulting in a temperature- and AFM thickness-dependent EB for nanostructures [172]. In the submicron disks of the same material system, Sort et al. found that the magnetization reversal mechanisms depended on the orientation of the external magnetic field, with exchange-biased vortices occurring for the magnetic field applied along the EB direction, while the vortex state was no longer reached when the angle between the applied field and EB direction became too large [173].

By embedding Ni nanoparticles in an IrMn matrix, an EB was observed up to a blocking temperature of around 400 K and a significantly increased coercive field, as compared to pure Ni nanoparticles [174]. Going one step further, Malinowski et al. combined IrMn with [Pt/Co]_3_ multilayers, in this way reaching perpendicular magnetization on polystyrene nanospheres with diameters of 58–320 nm [175]. They reported that the EB shift increased with the inverse of the sphere diameter, which they explained by a statistical distribution of Mn spins at the Co/IrMn interface.

Another interesting composition containing Mn is Mn_54_Al_46_, in which Ma et al. observed ferro- and antiferromagnetic phases in nanoribbons, resulting in EB, which was maximized at a cooling field of 41 kOe [176]. For nearly the same nanostructured system, i.e., Mn_55_Al_45_, a blocking temperature of 95 K was reported, as well as large EB shifts of 13 kOe at 10 K [177].

There are also different manganese oxides under investigation in EB systems. Li et al. observed an EB due to uncoupled surface spins in α-MnO_2_ rectangular nanowires, with a small positive EB above 18 K [178]. Only a small EB was found in ferrimagnetic/antiferromagnetic Mn_3_O_4_/MnO nanoparticles, but combined with a large coercive field, approximately three times larger than in bulk Mn_3_O_4_ [179]. In MnO/Mn_3_O_4_ core/shell nanoparticles with a diameter of 5–60 nm, EB and coercivity were observed to vary with the core diameter [180]. In similar nanoparticles, Si et al. also observed an increased Curie temperature as compared to bulk Mn_3_O_4_ [181].

**Figure 12:**
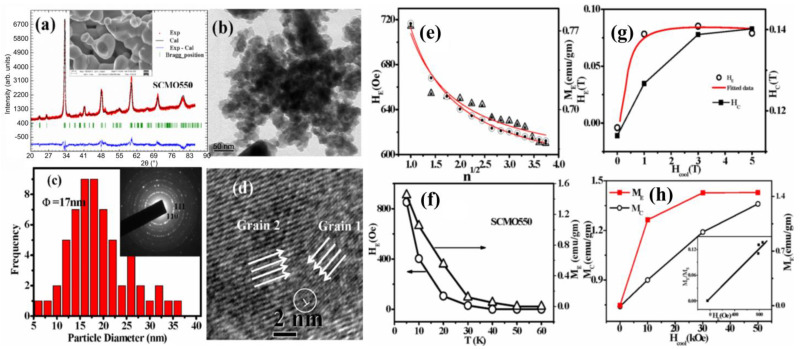
(**a**) Rietveld refinement (high-resolution diffraction, HRXRD) of X-ray diffraction (XRD) patterns of Sm_0.5_Ca_0.5_MnO_3_ nanoparticles. Inset shows an SEM image of these nanoparticles; (**b**) TEM micrographs; (**c**) histogram showing the size distribution of the nanoparticles, inset showing the selected area electron diffraction (SAED) pattern; (**d**) TEM image of a single particle (single crystalline), the circle marks a unit cell measurement; (**e**) EB field *H_E_* (circles) and vertical shift *M_E_* (triangles) (open symbols) as a function of the number of field cycles *n* at 5 K after FC in 7 kOe magnetic field. The solid lines show the best fitting with Binek’s relaxation equation. The solid symbols show that the data originated from this relaxation equation; (**f**) variation in *H_E_* (circles) and *M_E_* (triangles) with the temperature after FC in a 7 kOe magnetic field; (**g**) *H_E_* and *H_C_* vs. cooling field (H_cool_); (**h**) *M_E_* and *M_C_* vs. cooling field plot. Adapted from [185], originally published under a Creative Commons Attribution 3.0 Unported License.

In the manganite compound Pr_0.5_Ca_0.5_MnO_3_, antiferromagnetism was suppressed for nanostructures with diameters lower than 40 nm, while ferromagnetic clusters occurred with reduced sizes, resulting in an EB, which was maximized for an 85 nm particle diameter [182]. Similarly, in La_0.2_Ca_0.8_MnO_3_ nanoparticles with an average diameter of 15–37 nm, both horizontal and vertical loop shifts were observed, which strongly changed at a critical particle diameter of around 23 nm [183]. In similar nanoparticles of La_0.25_Ca_0.75_MnO_3_, prepared with diameters of 40–1000 nm, EB, vertical magnetization shift, and coercivity showed a maximum value at a diameter of approximately 80 nm at 5 K [184]. The authors reported a linear correlation between the EB and vertical shift, which they attributed to uncompensated spins being responsible for the EB in this manganite. Antiferromagnetic Sm_0.5_Ca_0.5_MnO_3_ nanoparticles (Figure 12a–d) displayed weak ferromagnetism below a temperature of 65 K and a spin glass transition below 41 K [185]. In this system, Giri et al. observed a training effect, which could be fitted by the Binek relaxation equation, as shown in Figure 12e, a sign change in the EB depending on the cooling field (Figure 12f), a strong temperature dependence of the EB (Figure 12g), and a cooling field dependence of the vertical shift *M_E_* and magnetic coercivity *M_C_* (Figure 12h) [185]. An EB caused by the interaction between the antiferromagnetic core and ferromagnetic shell was observed in CaMnO_3−δ_ nanoparticles [186]. Similarly, hexagonal YMnO_3_ nanoparticles presented an EB based on the exchange coupling between the antiferromagnetic core and spin glass shell or ferromagnetic surface spins, depending on the cooling field [187].

### 12.3. Exchange-Biased Nanostructures Containing Other Materials

In addition to the aforementioned materials, only a few other magnetic materials were used in the nanostructured exchange bias systems. One of them was the antiferromagnetic CuO, which Díaz-Guerra et al. synthesized as nanowires, with the cores showing antiferromagnetic behavior, while the shells behaved similar to a spin glass with uncompensated surface spins, resulting in a temperature and cooling field-dependent EB [188].

In nanocrystalline CoCr_2_O_4_, Goswami et al. observed an unconventional EB, which vanished above the spiral ordering temperature of the samples, indicating that the EB was based on the interaction between the collinear ferrimagnetic and spiral spin orders [189].

A highly unusual temperature-dependent magnetic behavior was observed in La_0.2_Ce_0.8_CrO_3_ nanoparticles, in which not only the EB shift, but also the magnetization changed its sign, which was explained by the interaction between the magnetization from the disordered surface shell and antiferromagnetic core [190]. Similarly, Lei et al. reported an EB and magnetization reversal in many rare-earth orthochromites [191].

## 13. Pseudo-Exchange Bias

**Figure 13:**
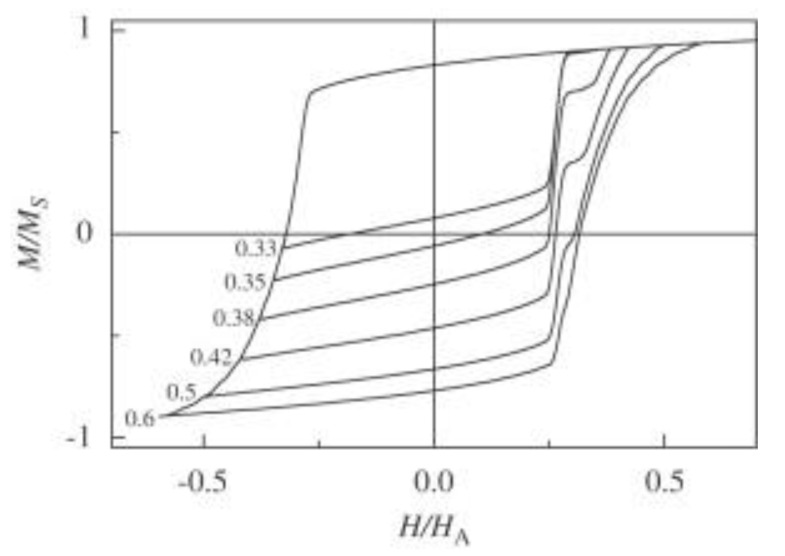
Minor hysteresis loops calculated for a disordered system of non-interacting single-domain particles with cubic anisotropy and different ratios (from 0.33 to 0.6). Reprinted with permission from [192]. Copyright 2008, Elsevier.

The exchange bias is recognized as a horizontal shift of the hysteresis loop, often connected with an additional vertical shift, sometimes showing open loops due to the training effect. However, all these effects can also be observed when minor loops (hysteresis loops in which saturation is not achieved in one or both field directions) are measured inadvertently, as previously mentioned [139]. The effect of minor loops was calculated exemplarily for a disordered system of non-interacting particles by Geshev, who discussed this problem with respect to another paper, and is shown in Figure 13 [192]. In this case, it is easily visible how even an asymmetry of the hysteresis loop can result from measuring minor loops, which may be interpreted as additional evidence for an EB system. Similarly, Geshev showed that the same effect could be responsible for the typical temperature-dependence of a horizontal shift, which could again be misinterpreted as being caused by EB.

This problem can especially occur in nanostructures in which the shape anisotropy may strongly block complete magnetization reversal, even if the maximum applied fields are 1 or 2 orders of magnitude larger than the coercive fields [193]. This pseudo-EB can be relatively small, especially in experiments in which hysteresis loops are averaged over a large number of nanostructures [194] but are nevertheless noticeable. It is even possible to tailor it by choosing suitable nanostructures [195,196].

While this pseudo-EB is fully suitable for diverse technological applications of EB systems and may, in some cases, even be an easier-to-produce alternative than EB systems, the physical effects should nevertheless not be confused.

## 14. Conclusions

Exchange bias in nanostructures has been investigated for decades. After the first comprehensive review in 2005 [6], many new effects were found, new material systems were investigated, and theoretical insights into the potential mechanisms responsible for the exchange bias were improved. Nevertheless, as this review shows, there are still many effects that have been experimentally investigated without fully modeling them, and again new material systems with other effects can be expected to be determined in subsequent years.

In addition to the horizontal shift opposite to the cooling field direction, which is the most common effect in exchange bias systems, there are several features occurring only in specific materials or material systems:

- A positive EB, i.e., a horizontal shift opposite to the usual direction, was modeled [48,49,50] and simulated through Monte Carlo simulations [62]. Experimentally, it was found, e.g., in NiO/Fe_3_O_4_ core–shell nanoparticles [120], nanostructured FeMn/Co/FeMn networks [160], and α-MnO_2_ rectangular nanowires [178].
- A vertical shift of the hysteresis loop was often observed, in many cases relatively small and negligible for the evaluation of the coercive fields, but sometimes strikingly large. This effect was simulated, e.g., in the domain state model [41,42,43,44] or in other Monte Carlo simulations [59,61]. Experimentally, it was observed in Co/CoO nanostructures [97], Co_3_O_4_ nanowires [112], milled Fe/MnO_2_ containing 20% Fe [148], and in Co_1−*x*_Ni*_x_*Fe_2_O_4_ [151] and Co_0.33_Fe_0.67_O nanoparticles [154], but also in manganite compounds, such as La_0.2_Ca_0.8_MnO_3_ [183], La_0.25_Ca_0.75_MnO_3_ [184], and Sm_0.5_Ca_0.5_MnO_3_ [185].
- The exchange bias shift was usually higher at lower temperatures and, in some cases, described as especially large, e.g., for aligned Co/CoO core–shell nanowires systems [79], core–shell Co/CoO nanoparticles [98], Fe_3−δ_O_4_@CoO core–shell nanoparticles [107], Co_3_O_4_ nanorods [113], Ni/NiO core/shell particles [115], Fe_3_O_4_/FeO core/shell nanoparticles [130], Fe/γ-Fe_2_O_3_ core–shell nanoparticles [135], or Zn_0.3_Ni_0.7_Fe_2_O_4_ as an example for a single-phase system [156].
- Many exchange bias systems showed asymmetric hysteresis loops, often interpreted as different magnetization reversal mechanisms on either side of the loop, which could be modeled in the Monte Carlo simulations [58,59] and were found in diverse EB systems, such as Co/CoO core/shell particles [99] or FeO/Fe_3_O_4_ core/shell nanoparticles [130].
- Surprisingly, an exchange bias could also be observed in some single-material systems, e.g., to interface layers forming between the substrate and material grown on it or the surface layers with different magnetic properties than the “bulk”. As some examples, CoFe_2_O_4_(111) grown on an Al_2_O_3_(0001) substrate can be mentioned [70], CoO nanoparticles [111], Co_3_O_4_ nanowires [112], Fe_3_O_4_ nanoparticles attached to Au seed particles [133], or Zn_0.3_Ni_0.7_Fe_2_O_4_ nanoparticles [156].
- Depending on the measurement, unrecognized minor loops can be misinterpreted as an exchange bias [192]. This “pseudo-EB” occurred especially often in nanostructures which, due to their large shape anisotropy, were prone to magnetic moments being blocked in a defined orientation [193,194,195,196].

Similar to the exchange bias effect in thin film systems [4], the exchange bias in nanostructures is still a highly important field of research, not only to achieve a basic understanding of the effects in different material combinations, but also for the increasing technological interest in spintronics devices. This review provided an overview of recent studies and may stimulate additional studies in this field of research.

## Data Availability

No data were created for this review paper.
